# Supplementary material for: Polyketide synthase gene clusters in the ericoid mycorrhizal fungus Oidiodendron maius: genome-wide prediction, expression profiling, and functional characterization of knockout mutants
Source: Front Plant Sci. 2026 Sep 16;17:1924430. doi: 10.3389/fpls.2026.1924430 (PMC13623903; doi:10.3389/fpls.2026.1924430)
Supplement: Supplementary file 1 [file SupplementaryFile1.zip › Supplementary Materials/Supplementary Folder 1.zip!/Oidma1_AssemblyScaffolds_Repeatmasked/knownclusterblast/cluster49/jgi.p_Oidma1_45889_mibig_hits.html]

| MIBiG Protein | Description | MIBiG Cluster | MiBiG Product | % ID | % Coverage | BLAST Score | E-value |
| --- | --- | --- | --- | --- | --- | --- | --- |
| EAL89337.1 | O-methyltransferase,\_putative | BGC0001403 | Trypacidin\_biosynthetic\_gene\_cluster | 48 | 102.286902287 | 470 | 1.8e-132 |
| AAD32742.2 | MmcR | BGC0000915 | Mitomycin\_biosynthetic\_gene\_cluster | 27 | 75.6756756757 | 110 | 4.8e-24 |
| ACH72896.1 | AflO | BGC0000011 | Aflatoxin/sterigmatocystin\_biosynthetic\_gene\_cluster | 27 | 69.6465696466 | 100 | 5e-21 |
| AAS90103.1 | OmtB | BGC0000006 | Aflatoxin\_biosynthetic\_gene\_cluster | 25 | 71.7255717256 | 93 | 8e-19 |
| AAS90012.1 | OmtB | BGC0000007 | Aflatoxin\_biosynthetic\_gene\_cluster | 24 | 75.2598752599 | 93 | 8e-19 |
| AAS90034.1 | OmtB | BGC0000008 | Aflatoxin\_biosynthetic\_gene\_cluster | 24 | 71.7255717256 | 92 | 1.8e-18 |
| AAS90060.1 | OmtB | BGC0000009 | Aflatoxin\_biosynthetic\_gene\_cluster | 24 | 73.1808731809 | 90 | 6.7e-18 |
| AAS90080.1 | OmtB | BGC0000010 | Aflatoxin\_biosynthetic\_gene\_cluster | 24 | 71.7255717256 | 89 | 1.5e-17 |
| AFD30960.1 | CrmM | BGC0000966 | Caerulomycin\_A\_biosynthetic\_gene\_cluster | 23 | 70.0623700624 | 84 | 3.7e-16 |
| AHV78244.1 | LasM | BGC0001245 | Lasiodiplodin\_biosynthetic\_gene\_cluster | 25 | 75.2598752599 | 76 | 1e-13 |
| AAL33762.1 | putative\_methyltransferase | BGC0000421 | Safracin\_biosynthetic\_gene\_cluster | 28 | 39.501039501 | 74 | 5e-13 |
| CAM34360.1 | putative\_O-methyltransferase | BGC0000242 | Lysolipin\_biosynthetic\_gene\_cluster | 31 | 35.1351351351 | 71 | 4.2e-12 |
